# Hospital accreditation: an umbrella review

**DOI:** 10.1093/intqhc/mzad007

**Published:** 2023-02-04

**Authors:** Katherine Lewis, Reece Hinchcliff

**Affiliations:** School of Public Health & Social Work, Queensland University of Technology, Kelvin Grove, QLD 4059, Australia; Oral Health, Sunshine Coast Hospital and Health Service, Queensland 4059, Australia; School of Public Health & Social Work, Queensland University of Technology, Kelvin Grove, QLD 4059, Australia; Centre for Health Management, Faculty of Health, University of Technology Sydney, Sydney, NSW 4059, Australia

**Keywords:** accreditation, quality improvement, quality assessment, patient safety, external evaluation, quality standards

## Abstract

Hospital accreditation is an established quality improvement intervention. Despite a growing body of research, the evidence of effect remains contested. This umbrella review synthesizes reviews that examine the impacts of hospital accreditation with regard to health-care quality, highlighting research trends and knowledge gaps. Terms specific to the population: ‘hospital’ and the intervention: ‘accreditation’ were used to search seven databases: CINAHL (via EBSCOhost), Embase, Medline (via EBSCOhost), PubMed, Scopus, the Cochrane Database of Systematic Reviews, and the Joanna Briggs Institute (JBI) EBP Database (via Ovid). 2545 references were exported to endnote. After completing a systematic screening process and chain-referencing, 33 reviews were included. Following quality assessment and data extraction, key findings were thematically grouped into the seven health-care quality dimensions. Hospital accreditation has a range of associations with health system and organizational outcomes. Effectiveness, efficiency, patient-centredness, and safety were the most researched quality dimensions. Access, equity, and timeliness were examined in only three reviews. Barriers to robust original studies were reported to have impeded conclusive evidence. The body of research was largely atheoretical, incapable of precisely explaining how or why hospital accreditation may actually influence quality improvement. The impact of hospital accreditation remains poorly understood. Future research should control for all possible variables. Research and accreditation program development should integrate concepts of implementation and behavioural science to investigate the mechanisms through which hospital accreditation may enable quality improvement.

## Introduction

A prominent method of improving hospital quality is accreditation, involving assessments of compliance against predetermined standards [1, 2]. Considering a valid indicator of a high-performing organization [3], accreditation programs are established globally with both voluntary and mandatory models [1, 2, 4–6]. Assessments are undertaken by government or independent organizations [7] and may encompass health organisations or individual hospitals or be speciality-specific [4, 6]. Accreditation standards cover domains such as clinical governance and patient-centredness [1, 8], with the consequences of failing to meet these standards variable across different health system contexts [7].

While it has been suggested that positive relationships exist between accreditation and organizational management, outcomes, and quality indicators [9, 10], concerns remain that this relationship is not causal [1, 9] or sustained [11]. Critics have identified that safety failures still occur in accredited hospitals [1], and the extent to which accreditation influences clinical and organizational performance is poorly understood [3].

This is the first umbrella review to examine and synthesize the results of existing reviews on hospital accreditation. The aim was to establish the effectiveness of accreditation programs, both mandatory and voluntary, on improving hospital quality. Research trends and methodological approaches in the literature were highlighted, and future research directions were recommended. The framework in Table 1 was used to present the results. Developed by Araujo *et al.*, it defines seven health-care quality dimensions [12] using established concepts [13–15].

**Table 1.** The seven health-care quality dimensions [12].

Health care quality dimension	Description
Effectiveness	The health-care service is delivered based on scientific knowledge and results in improved health-care outcomes. Health services are provided to all who could benefit, refraining from services to those not likely to benefit.
Efficiency	The health-care service is delivered in a manner, which maximizes resource use and avoids waste, including waste of equipment, supplies, ideas, and energy. It aims the greatest health improvement at the lowest cost, with the most advantageous cost–benefit.
Access	The health care is timely, geographically reasonable, and provided in a setting where skills and resources are appropriate to medical need.
Patient-centredness	The health care is respectful of and responsive to individual patient preferences, needs, culture, and values. There is conformity to patient preferences regarding the patient–practitioner relationship, the service accessibility and amenities, and the effects and costs of care.
Equity	The health care does not vary in quality because of personal characteristics such as gender, ethnicity, geographic location, and socioeconomic status. It accounts for fairness in the distribution of care and its effects on health.
Timeliness	The health care is delivered in a timely manner, reducing waiting times and harmful delays for both those who receive and those who give care.
Safety	The health care is delivered in a manner, which minimizes risk and harm to service users, avoiding injuries to patients from the care that is intended to help them.

### Review objectives

The key questions the review sought to answer are as follows:

- What is the evidence to support the effectiveness of hospital accreditation with regard to health-care quality?
- What are the research trends?
- What are the knowledge gaps that need to be addressed to enable evidence-informed improvements to hospital accreditation programs and research?

## Methods

This umbrella review is based on the JBI umbrella review methodology [16] and registered on Prospero (ID: CRD42021284015).

### Search strategy and selection criteria

Search terms were developed using the PICO framework. The population term was ‘hospitals’, and ‘accreditation’ was the intervention of interest. The comparison term: ‘no accreditation’ and the outcome term: ‘impact of accreditation’ were omitted as they were not considered to improve the sensitivity of the literature search.

Seven electronic bibliographic research databases were searched: CINAHL (via EBSCOhost), Embase, Medline (via EBSCOhost), PubMed, Scopus, the Cochrane Database of Systematic Reviews, and the JBI EBP Database (via Ovid). Aligning with the JBI methodology, an additional search using the terms ‘literature review’, ‘systematic review’, ‘scoping review’, ‘narrative review’, ‘rapid review’, and ‘meta-analysis’ was used to limit the results to studies that used these methodologies. The search was completed on 30 October 2021, and 2545 articles were exported to endnote for screening. The search strategy can be found in Supplementary File 1.

To include all relevant reviews of hospital accreditation, a two-step process was undertaken. Step one excluded reviews that exclusively examined nonhospital settings. Step two discerned between reviews that combined both hospital and other health-care settings to include only reviews where the findings relevant to hospital settings could be determined. Both qualitative and quantitative reviews were included, as were reviews of mandatory, voluntary, and the Magnet Recognition Program [17]. Only published reviews where the full text was available from within the authors’ academic institution were included. Accepting the risk of omitting key literature, primary research and non-English language studies were excluded. No location or date restrictions were applied to identify a broad range of global literature and research trends over time. Chain-referencing of included studies was undertaken to maximize the number of included studies.

**Figure 1:**
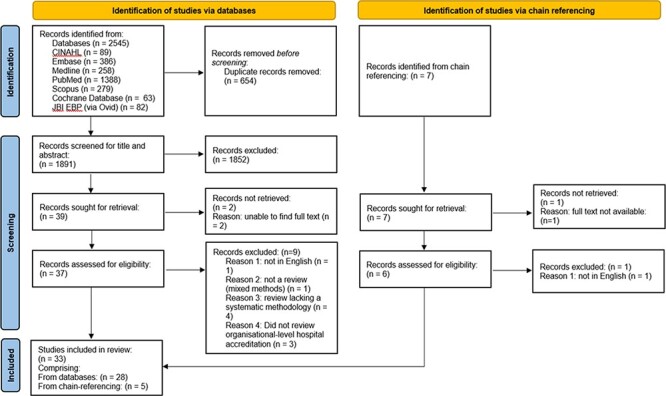
PRISMA flowchart.

Title and abstract screening was led by K.L. Ten percent of identified studies at the title and abstract screening, full-text screening, quality assessment, and data extraction stages were sampled by R.H. Differences in opinion were resolved through discussion, with agreement that the sample size of 10% was acceptable. The process is presented in the PRISMA flowchart [18] in Fig. 1.

### Assessment of methodological quality

Following the JBI Umbrella Review Methodology, the JBI Critical Appraisal Checklist for Systematic Reviews and Research Syntheses [16] was used and included a risk of bias assessment. The results can be found in Supplementary File 2. No studies were excluded due to quality issues.

### Data extraction

Data were extracted in three tables that can be found in Supplementary File 3. To identify research trends, Supplementary Table S2 presents study characteristics: author and year, title, methodology, inclusion and exclusion criteria, number of included studies, and source of publication. As presented in each review, Supplementary Table S3 presents review questions, key findings, and implications for practice and research, and Supplementary Table S4 presents findings related to the seven health-care quality dimensions.

## Results

### Characteristics of included studies

Thirty-three reviews were included. The earliest was published in 2006 [19], and the number of reviews published each year has been increasing. The International Journal for Quality in Health Care was the most common publication (four reviews) [12, 20–22].

Differences in methodologies were apparent with 10 systematic reviews [12, 20, 23–30], three scoping reviews [31–33], two integrative reviews [34, 35], and one meta-analysis [36]. The remaining 17 reviews employed a systematic search strategy and a narrative synthesis of findings [19, 21, 22, 37–50].

Three studies reviewed hospital accreditation research [20, 25, 43], and three reviewed accreditation policy [19, 22, 47]. Two reviewed hospital accreditation implementation [44, 48], and one specifically reviewed cost benefits [21]. Three systematic reviews were updated [20, 24, 41].

Reviews were varied with regard to quality, but this did not appear to influence major differences in study conclusions. Three reviews met only one of the quality criteria, with two of these searching only one database and not controlling for errors in data extraction and critical appraisal [39, 46]. Allowing for nonapplicable quality criteria, all three scoping reviews returned the maximum quality scores and followed Arksey and O’Malley’s scoping review framework [51]. Greater variation was observed between the systematic reviews, with only five achieving the maximum quality score. Bias was not consistently manageable, with qualitative syntheses being the dominant approach.

Individual studies were included in multiple reviews. For example, fourteen reviews included the same randomized controlled trial [19–21, 23, 25, 26, 37, 38, 41–45, 48], which returned mixed bias assessments from two separate reviews [25, 42]. Furthermore, one systematic review included four studies, two of which were systematic reviews that were also included in this study [25, 37, 41].

One review could not identify any studies that met its inclusion criteria [30]. The most studies included by a review were 122 [43]. This represents a wide variation in hospital accreditation review methodologies and indicates that the trend of heterogeneity in primary accreditation research [25, 35, 36, 43] is also present in accreditation reviews. A wide heterogeneity also became evident in the exploration of impacts of specific programs, with some reviews lacking the impact of specific programs, while others reviewed all programs within a specific country or jurisdiction.

The included review findings were of unequal proportion across the seven quality dimensions. Five reviews did not demonstrate any findings specifically related to the health-care quality dimensions [30, 31, 41, 42, 45]. The four quality dimensions represented most often were effectiveness, efficiency, patient-centredness, and safety, with a minority of findings in the equity, access, and timeliness dimensions.

### Health care quality dimensions

#### Effectiveness

Seventeen reviews provided results related to effectiveness, with 10 proposing positive links [12, 19, 24, 26, 33, 36, 37, 39, 49, 50], citing accreditation’s promotion of evidence-based practice [19, 33], guideline development [37, 39], and increased use of clinical indicators [39] and data [33].

Specific outcomes considered to have improved due to accreditation include length of stay [12, 26, 49, 50], readmission rates [12], and mortality rates [12, 36, 49]. However, a meta-analysis of bariatric surgery outcomes recommended that mortality rate reductions should be interpreted with caution, due to the heterogeneous nature of the included studies and the facilities from which its data were collected [36].

The remaining five reviews that addressed effectiveness were unable to draw any conclusions regarding positive impacts [20, 22, 27, 28, 43].

#### Efficiency

Nineteen reviews provided efficiency results with two broad conflicting themes. Reported positive impacts were related to staff retention and lower turnover in accredited hospitals [12, 28, 32, 34, 35, 43, 49]. It has been suggested that this is associated with increased employee satisfaction resulting from accreditation processes [43] and reduced burnout [49]. The authors of one review reported that accreditation is perceived by health professionals to reduce costs and increase efficiency [33]. This may be related to other positive links, such as improved teamwork [40] and productivity [26, 40]. Other authors associated hospital accreditation with improved resource management [12, 19, 24].

In contrast, one review was not convinced that accreditation programs impact efficiency [29]. It was also reported that the administration of hospital accreditation can introduce inefficiencies through increased costs and workload, divert clinicians away from providing direct patient care [19, 25–27, 38, 43, 44, 50], and demand investments in extra equipment to meet standards [26]. Overall, due to a lack of rigorous economic studies to examine costs versus benefits [21, 28, 43], the true impact on efficiency has been deemed inconclusive [21].

#### Access

The relationship between accessibility and hospital accreditation was poorly represented in the literature. Two reviews provided results related to accessibility, with one using quantitative data to conclude that accreditation has a ‘null’ effect [12]. Hospital accreditation has also been used as a lever to promote universal health coverage in low- and middle-income countries, where scarcity of resources shifts the focus of health system quality to improving accessibility of services [47].

#### Patient-centredness

Eighteen reviews provided results related to patient-centredness. Accreditation was found to be positive in its potential to boost public confidence [19, 44, 48], improving public disclosure [20], consumer involvement [23], complaints management [33], communication, infrastructure [40], and the working environments of accredited hospitals [34]. As these benefits may be used to attract patients, they may also be motivators to pursue accreditation [48].

However, most reviews that reported on patient-centredness found inconclusive, inconsistent, or no evidence of impacts on patient-centredness [12, 19, 20, 22, 26, 37, 43, 46, 50]. One review that specifically examined the association between accreditation and patient satisfaction found no relationship between the two [23], and one concluded that this area is relatively under-researched [20].

#### Equity

Equity was only examined by two reviews [12, 47]. One was unable to find any related primary research [12], with the other describing the use of hospital accreditation to promote equity in low- and middle-income countries [47].

#### Timeliness

Two reviews suggested that hospital accreditation has a positive effect on timeliness [12, 32]. However, one review’s findings were based on just two primary studies [12], and the second review associated Magnet Recognition with improved patient flow [32].

#### Safety

Conclusions regarding the impact of hospital accreditation on safety were mixed, with most of the 16 reviews that reported on safety finding inconclusive results [20, 22, 27, 28, 33, 43, 49]. Reviews that found a positive impact [12, 26, 37, 50] suggested that this was due to promoting an improved patient safety culture [26] and procedures [29] such as those used to prevent and manage falls [19, 40]. A safe organizational culture was considered by the authors to contribute to other findings, including a positive impact on safety indicators, increased incident reporting [26], and fewer adverse events [12, 19], such as postsurgery complications [24, 36] and infection rates [12, 19, 37].

### Additional impacts and issues

This review revealed a range of positive associations between hospital accreditation and health system and organizational outcomes [12, 19, 24, 26, 28, 32–34, 37, 40, 43, 46, 47, 49, 50]. Hospital accreditation is an accountability [21, 50], regulatory [47], quality assurance [19], quality improvement [47], marketing [28, 48], and financial incentive [48] instrument. In low- and middle-income countries, accreditation has been extended to shape the medical tourism industry and to drive macro-level policy [47]. Accreditation processes are considered suitable for hospital environments due to the inherently high risks of process failures and error [19] and as such can be used to strengthen public confidence [29], which, in turn, benefits hospital service providers [42]. The attainment of mandatory or voluntary accreditation reflects high organizational performance [35, 50] as it influences process development [33, 50], management [40], and patient safety culture [26]. Importantly, health-care quality is represented in the literature as a subjective concept [20], influenced by competing and contextually specific health system and cultural, economic, political, social, environmental, and professional factors [19, 27, 47, 48].

Hospital accreditation, however, is only a snapshot of quality assurance [19], and so concerns remain that any impacts are not sustained [22]. Participation in hospital accreditation programs is not without risk, which for mandatory programs may include service restrictions if expectations are not met [19]. All program types require substantial resource investment and significant change processes [34, 46]. The resource burden is greater for smaller organizations [19], and accreditation may be economically unsustainable in some low- and middle-income countries [47]. There is also concern that where inspections focus on standard attainment [44] rather than quality improvement that directly enhances the provision of clinical care [50], recommendations may only improve management and support systems [44]. The former ethos may explain accreditation’s association with increased workloads and stress [19, 25–27, 33, 38, 43, 44, 50].

A common recommendation of hospital accreditation reviews is for cost–benefit or cost-effectiveness analyses [12, 19–21, 25, 37, 42, 43, 47, 48]. In the present global environment, concerns of health system sustainability due to increasing complexity, costs, and public expectation [6, 52, 53] are significant drivers of the need for enhanced quality and safety [54]. Until accreditation benefits can be defined, pursuing a cost–benefit or cost-effectiveness analysis is difficult and the opportunity cost for health systems to retain accreditation as a vital quality improvement mechanism remains unknown [21].

### Research issues and challenges

Although clinical performance indicators intend to improve health-care quality [39], their use in accreditation research is limited [43]. This limits opportunities for empirical research, thus making it difficult to attribute improved clinical outcomes to accreditation [35, 43]. Primary research is also heterogeneous, often with poor methodological rigour [25, 35, 36], lacking in theory [45], and disproportionately low compared to the high costs of accreditation to governments and health-care organizations [43].

Research is also challenged by difficulties in isolating hospital accreditation impacts from concurrent influencing factors [25, 27, 36, 45, 47]. Limited control over potential interactions among variables [45, 50], the inherent complexities of accreditation programs [25, 31, 43], and their differing aims, focus, design, and maturity [29] means that programs are difficult to compare.

Only three of the included reviews explicitly considered the use of theory to interrogate, categorize, or explain the results from original studies. Two reviews used different theories in their study design: the policy transfer framework [47] and the Consolidated Framework for Implementation Research [44]. The authors of a third accreditation review proposed an accreditation research framework to facilitate the introduction of relevant theory, for heterogeneous research settings, with nonexperimental designs [45]. The inclusion of theory in these reviews not only demonstrated the aim of explaining how or why their findings occurred but also revealed a more generalized lack of theoretical basis in hospital accreditation research.

## Discussion

### Statement of principal findings

Consequent to mixed results, research limitations, methodological flaws, and theoretical deficiencies, the lack of conclusive evidence into the effectiveness of accreditation provides an impetus to continue the quest to determine causality and cost-effectiveness. The conceptual basis for improving quality and safety is established in defining ‘what’ accreditation is to achieve, and so improved patient outcomes should be the overall aims of accreditation programs and research. Review conclusions are typically generalized, continuing to question if accreditation ‘works’ and struggling with inherent heterogeneities. Where possible, overcoming these heterogeneities is vital and primary research and systematic reviews may yield more conclusive insights by investigating the impacts between or within jurisdictions, specific programs, types of assessments, and mandatory and voluntary accreditation.

Despite a lack of evidence, with perceived benefits to organizations, governments, and public confidence, hospital accreditation remains a popular policy, with voluntary accreditation attractive to high-performing, well-resourced organizations. Significant external influencing factors mean that accreditation programs may be more closely associated with the competing perspectives and needs of the specific health system in which it operates, than its impact on health-care quality. For instance, mandatory accreditation programs must be carefully designed, be reasonably achievable and not undermine wider health system objectives. This is because health systems are accountable for providing permanent access to health care, coverage, continuity, and efficiency [54], but this is at risk if sanctions following nonattainment result in service restriction, closure, or financial penalties. It is important to also note, however, that while the limited evidence base has not deterred expansions of accreditation globally, there are increasing examples where other approaches are being implemented [55]. An implication is that for accreditation to retain or expand its current profile, further efforts may be required to strengthen the evidence base.

The lack of evidence within the equity, access, and timeliness domains also raises questions regarding the foci of accreditation programs and research. There is a trend in Australia, for example, to accredit entire health services. As health services have a broader mission than individual hospitals, accreditation programs may require modification to ensure maximum relevance at that higher level of a health system, requiring focus on quality dimensions like access and equity.

### Interpretation within the context of the wider literature

The mechanisms explaining how hospital accreditation may improve health-care quality is poorly discussed in the literature. When considering the more general function of quality improvement activities as instruments of change [20, 45, 47], understanding how organizations and health-care professionals engage with accreditation processes could offer significant value in improving patient outcomes. Supportive systems have long been recommended to facilitate improved outcomes [56]; however, it is the health-care professionals who mediate and maintain improvements [57]. Research indicates that the uptake of evidence-based improvements by health-care professionals is poor, or not sustained [58], and that relationships between governance mechanisms and health professionals are not properly considered in their development [57]. As other research has found that understanding the contexts of the clinical microsystem is essential in facilitating the successful implementation of quality improvements [59], implementation theory is ideal to enrich both accreditation research and program development and to understand how hospital accreditation policy may successfully transcend across the complex and interdependent levels of a health system [60]. No review identified in this study thoroughly investigated how accreditation may have this effect. This persistent gap in knowledge may act as an additional influence on the scepticism of health-care professionals [37] who are required to engage positively for accreditation to impact clinical outcomes.

### Strengths and limitations

The exclusion of non-English language reviews, grey literature, and unpublished studies means that key literature may have been omitted. The review included findings from overlapping reviews, with examples of primary research and systematic reviews represented multiple times, possibly introducing biased or skewed results. Although the quality assessment did not result in the exclusion of studies, similar results and conclusions that were observed offered an opportunity to recommend alternative research directions. Due to the qualitative nature of most of the included reviews, the use of statistical tools to investigate heterogeneity was not feasible.

### Implications for policy, practice, and research

Policymakers, agencies, and health-care organizations should adopt an evidence-informed approach to implementing accreditation programs, focusing upon how programs may achieve improved patient outcomes. Rigorous methodologies integrating implementation science theories are recommended to understand the contexts in which hospital accreditation can make the most significant improvements. Outcome indicators should be included in the research design. Primary research and systematic reviews may also benefit from research questions that control for possible variables to identify the successful elements of accreditation programs.

## Conclusion

Hospital accreditation seems likely to remain a popular policy, appearing to develop and mature under external influences: economics, politics, and culture, more than an evidence base. It remains that there is insufficient evidence that hospital accreditation improves hospital quality and positive impacts should be interpreted with caution, considered only as associations. To prioritize individual and population outcomes, opportunities for theoretical development into how hospital accreditation may result in health-care quality improvement should be undertaken.

## Supplementary Material

mzad007_Supp

## Data Availability

No new data were generated or analysed in support of this review.
