# Supplementary material for: Hospital accreditation: an umbrella review
Source: Int J Qual Health Care. 2023 Feb 4;35(1):mzad007. doi: 10.1093/intqhc/mzad007 (PMC9950788; doi:10.1093/intqhc/mzad007)
Supplement: mzad007_Supp [file mzad007_supp.zip › suppl_data/Clean_Supplementary_file_3.docx]

Table 2: Study Characteristics

| **Author and year** | **Title** | **Methodology** | **Inclusion/ exclusion** | **No of included studies** | **Journal** |
| --- | --- | --- | --- | --- | --- |
| Alkhenizan & Shaw, 2011 [37] | Impact of accreditation on the quality of healthcare services: a systematic review of the literature | Systematic literature review | Inclusion: all studies that evaluated the impact of general or subspecialties accreditation programs on the quality of healthcare services. No language restrictions. | 26 | Annals of Saudi Medicine |
| Alkhenizan & Shaw, 2012 [38] | The attitude of health care professionals towards accreditation: A systematic review of the literature | Systematic literature review | Inclusion: all studies that had evaluated the attitude of health care professionals. No language restrictions. | 17 | Journal of Family and Community Medicine |
| Almasabi et al., 2014 [23] | A Systematic Review of the Association Between Healthcare Accreditation and Patient Satisfaction | Systematic review | Inclusion: English language studies which focused on health organisation accreditation and presented information on the relationship with patient satisfaction. | 20 | World Applied Sciences Journal |
| Almoajel, 2012 [39] | Relationship between accreditation and quality indicators in hospital care: A review of the literature | Systematic literature review | Inclusion: English language studies that focus on perspectives at national level on international and local experience on accreditation.  Exclusion: focus on educational and training programs; the effect of accreditation to clinical or perspective outcomes; disease-oriented focus; specific treatment modalities; specific specialties; health workforce perspectives | 23 | World Applied Sciences Journal |
| Anderson et al., 2018 [34] | Impact of MAGNET hospital designation on nursing culture: an integrative review | Integrative literature review | Inclusion: English language studies of all methodologies, that researched Magnet hospitals in the process of designation and first-time Magnet hospitals. | 29 | Contemporary nurse |
| Araujo et al., 2020 [12] | Hospital accreditation impact on healthcare quality dimensions: a systematic review | Systematic review | Inclusion: Studies in English/ Portuguese that empirically assess the impact of hospital accreditation on Healthcare Quality Improvement, through quantitative methods.  Exclusion: review papers, analysis of perceptions, articles dealing with accreditation for specific services. | 36 | International Journal for Quality in Healthcare |
| Avia & Hariati, 2019 [40] | Impact of hospital accreditation on quality of care: A literature review | Literature review | Inclusion: quantitative and qualitative studies that discuss/ evaluate the impact of hospital accreditation on the quality of care.  Exclusion: studies that only evaluate the accreditation program, or only evaluate one specialty. Studies that are not in Indonesian/ English | 20 | Enfermeria Clinica |
| Azagury et al., 2016 [24] | Bariatric Surgery Outcomes in US Accredited vs Non-Accredited Centers: A Systematic Review | Systematic review | Inclusion: English language articles comparing accredited with non-accredited centres | 13 | Journal of the American College of Surgeons |
| Baidwan et al., 2020 [36] | A meta-analysis of bariatric surgery-related outcomes in accredited versus unaccredited hospitals in the United States | Systematic review with Meta-analysis | Inclusion: English language studies that specifically evaluated the impact of accreditation status on bariatric surgery-related outcomes, that is, studies that compared the unaccredited centres with the accredited centres or compared healthcare centres pre- and post- National Coverage Decision with regards to the outcomes of interest. | 13 | Clinical Obesity |
| Brubakk et al., 2015 [25] | A systematic review of hospital accreditation: the challenges of measuring complex intervention effects | Systematic review | Inclusion: full-text publications that evaluated the impact of overall hospital accreditation programs on the quality of healthcare services from January 2000 – February 2020: systematic reviews, randomised controlled trials, non- randomised controlled trials, controlled before and after studies, interrupted time series. No language restrictions | 4 | BMC Health Service Research |
| Cerqueira, 2006 [19] | A literature review on the benefits, challenges, and trends in accreditation as a quality assurance system | Systematic literature review | Inclusion: Studies examining the benefits and/ or challenges and/ or current trends in accreditation | 21 | Thesis |
| Danno et al., 2021 [35] | Quality improvement programs and the professional nursing practice environment: an integrative review | Integrative review | Inclusion: English/ Portuguese/ Spanish language studies: original surveys, with the application of a term of consent and validated instruments in the nursing staff at hospitals which have a consolidated quality improvement program | 10 | Revista Brasileira de Enfermagem |
| Flodgren et al., 2011 [41] | Effectiveness of external inspection of compliance with standards in improving healthcare organisation behaviour, healthcare professional behaviour or patient outcomes | Systematic literature | Inclusion: studies evaluating the effect of external inspection against external standards on healthcare organisation change, healthcare professional behaviour or patient outcomes in hospitals, primary healthcare organisations and other community-based healthcare organisations. We considered the following study designs: randomised controlled trials, controlled clinical trials, interrupted time series and controlled before and after studies that included at least two sites in both control and intervention groups.  No language restrictions | 2 | Cochrane review |
| Flodgren et al., 2016 [42] | External inspection of compliance with standards for improved healthcare outcomes | Systematic literature | Inclusion: randomised controlled trials, non‐ randomised controlled trials, interrupted time series and controlled before and after studies evaluating the effect of external inspection against external standards on healthcare organisation change, healthcare professional behaviour or patient outcomes in hospitals, primary healthcare organisations and other community‐based healthcare organisations. No language restrictions. | 2 | Cochrane review |
| Gamble et al., 2021 [31] | Hospital accreditation: Driving best outcomes through continuity of midwifery care? A scoping review | Scoping | Inclusion: English language articles, policies, reports and guidelines on hospital accreditation in Australia, with emphasis on continuity of midwifery care, at the 'macro' level | 100 | Women Birth |
| Greenfield & Braithwaite, 2008 [20] | Health sector accreditation research: a systematic review. | Systematic review | Inclusion: English language empirical work that systematically examined accreditation or the accreditation process; how it works, what it does, results, surveyors and their processes. | 66 | International Journal for Quality in Healthcare |
| Hinchcliff et al., 2012 [43] | Narrative synthesis of health service accreditation literature | Systematic review with narrative synthesis | Inclusion: References had to focus on one or more aspect of health service accreditation programs or processes and be of empirical research. English language studies.  Exclusion: research into professional development or medical credentialing programs, non-systematic literature reviews and commentaries | 122 | BMJ Quality and Safety |
| Hovlid et al., 2020 [44] | Mediators of change in healthcare organisations subject to external assessment: a systematic review with narrative synthesis | Systematic review with narrative synthesis | Inclusion: English/ Norwegian/ Swedish or Danish language quantitative and qualitative studies about external inspections that include empirical data about mediators of change at an organisational level for care delivery. | 95 | BMJ Open |
| Hussein et al., 2021 [26] | The impact of hospital accreditation on the quality of healthcare: a systematic literature review | Systematic literature | Inclusion: quantitative publications that evaluated the impact of overall hospital accreditation programs on the quality of health care in the last two decades. No language restriction. Excluded: review articles. | 76 | BMC Health Service Research |
| Johnston et al., 2020 [32] | What impact does Magnet designation have on emergency department nurses' outcomes? A scoping review | Scoping | Inclusion: a) experimental/ observational/ qualitative/ opinion pieces or case studies b) the impact of Magnet status on nurses' outcomes c) the study was conducted in Emergency Departments or trauma setting.  Exclusion: Incomplete reports and non-peer reviewed literature. Non – English language studies | 3 | International Emergency Nursing |
| Khan et al., 2021 [33] | The impact of hospital accreditation in selected Middle East countries: a scoping review | Scoping | Inclusion: English/ Arabic language studies that were  1) original research based on primary data collection/ review of existing registry data 2) focussed on hospital accreditation programmes in Iran/ Jordan/ Saudi Arabia 3) evaluated any types of impacts of hospital accreditation programmes | 41 | Journal of Health Organization and Management |
| Kilsdonk et al., 2015 [45] | Evaluating the impact of accreditation and external peer review | Systematic literature | Inclusion: Original research on the impact of accreditation or external peer review on quality-related outcome measures. English/ Dutch language studies  Exclusion: settings that do not deliver direct patient care | 50 | International Journal of Health Care Quality Assurance |
| Lazzeri et al., 2019 [46] | Accreditation and quality in the Italian national health care system: A 10 year's long review | Literature review | Inclusion: English/ Italian original articles, (but also letters to the editor and short communications if containing original data) that reported clear data on: i) Italian region or city in which the study was conducted; ii) number of centres involved in the study; ii) original and interesting results derived from the experiences of accreditation | 16 | Epidemiology Biostatistics and Public Health |
| Mansour et al., 2020 [47] | The development of hospital accreditation in low- and middle-income countries: a literature review | Literature review | Inclusion: qualitative and quantitative studies of any design, that looked at hospital accreditation and its development in any low- and middle- income countries (LMIC) or group of LMICs.  Exclusion: any study that focussed on accreditation in any non-hospital setting. Non-English language studies | 78 | Health Policy and Planning |
| Mumford et al., 2013 [21] | Health services accreditation: what is the evidence that the benefits justify the costs? | Literature review | Inclusion: English language empirical research studies on the topic of economic evaluation of health service accreditation, and analytical studies that defined, or quantified costs | 21 | International Journal for Quality in Health Care |
| Ng et al., 2013 [48] | Factors affecting the implementation of accreditation programmes and the impact of the accreditation process on quality improvement in hospitals: a SWOT analysis. | Systematic literature | Inclusion: English language comparative observational or qualitative studies into the implementation of hospital accreditation programmes, and their outcomes.  Exclusion: reviews, periodicals, and conference reports; articles on single specialty accreditation programmes. | 26 | Hong Kong Medical Journal |
| Petit dit Dariel & Regnaux, 2015 [27] | Do Magnet®-accredited hospitals show improvements in nurse and patient outcomes compared to non-Magnet hospitals: a systematic review | Systematic review | Inclusion: Quantitative studies comparing nurse and patient outcomes in Magnet and non-Magnet hospitals.  When these were not available case-controlled, descriptive comparative and descriptive correlational designs were considered. | 10 | JBI Database of Systematic Reviews and Implementation Reports |
| Rodríguez-García et al., 2020 [49] | How Magnet Hospital Status Affects Nurses, Patients, and Organizations: A Systematic Review | Systematic review | Inclusion: English/ Spanish language original comparative studies exploring outcomes in Magnet and non-Magnet hospitals or in Magnet, Magnet-aspiring, and non-Magnet hospitals were included. No restriction in regard to study design | 21 | The American Journal of Nursing |
| Salmond et al., 2009 [28] | A comprehensive systematic review of evidence on determining the impact of Magnet designation on nursing and patient outcomes: is the investment worth it? | Systematic review | Inclusion: Any quantitative or qualitative English language study comparing organisational, nurse, patient or economic outcomes in Magnet designated hospitals with a comparison to a non-Magnet facility | 17 | JBI Library of Systematic Reviews |
| Swathi et al., 2020 [50] | Impact of accreditation on performance of healthcare organizations: A review of global studies | Literature review | Inclusion: English language empirical research, that focussed on one or more aspects of the effect of health care accreditation | 62 | International Journal of Quality and Service Sciences |
| Tabrizi et al., 2011 [29] | Advantages and Disadvantages of Health Care Accreditation Models | Systematic review | Inclusion: English/ Farsi language articles that assessed accreditation models and considered their advantages and disadvantages | 83 | Health Promotion Perspectives |
| Van Wilder et al., 2021 [22] | Is a hospital quality policy based on a triad of accreditation, public reporting and inspection evidence-based? A narrative review | Narrative literature review | Inclusion: English/ Dutch language original quantitative research from high- or middle- income countries concerning secondary or tertiary care | 69 | International Journal for Quality in Health Care |
| Vist et al., 2009 [30] | Effect of Certification and Accreditation of Hospitals | Systematic review | Inclusion criteria: Randomised Controlled Trials, controlled before and after studies, interrupted time series. Studies of all types of hospitals as long as the intervention included certification or accreditation | 0 | NIPH Systematic Reviews: Executive Summaries |

Table 2

Table 3: Key findings

| **Author and year** | **Review questions/ aims/ objectives** | **Key findings** | **Practice recommendations** | **Research recommendations** |
| --- | --- | --- | --- | --- |
| Alkhenizan & Shaw, 2011 [37] | To evaluate the impact of accreditation programs on the quality of healthcare services. | Accreditation supported | Educate healthcare professionals about the potential benefits of accreditation to resolve any sceptical attitude of healthcare professionals towards accreditation. Accreditation programs should be supported as a tool to improve the quality of healthcare services | Nil |
| Alkhenizan & Shaw, 2012 [38] | To review the literature on the attitude of health care professionals towards accreditation. | Generally, attitudes are good, however findings are not consistent. Costs persist as a barrier to perceived value. | Healthcare professionals (especially physicians) have to be educated on the potential benefits of accreditation. Accreditation can be used as a marketing tool. | The concern of leaders of health care organizations was also that the benefits of accreditation might not be worth the cost and the effort involved in the process. These concerns can only be addressed by means of a rigorous cost-benefit analysis. |
| Almasabi et al., 2014 [23] | To understand the relationship between accreditation and patient satisfaction | No significant relationship between patient satisfaction and accreditation | Patient satisfaction should be on the agenda to improve accreditation programs | Multi method approach required to understand complexity of accreditation |
| Almoajel, 2012 [39] | To identify the relationship between accreditation and quality indicators used in hospital care services | Inconsistent results, unable to decisively attribute accreditation to improvements in indicators | Nil | Nil |
| Anderson et al., 2018 [34] | Does the process of attaining MAGNET designation impact organisational culture for nurses and if so, how? | MAGNET framework is supported | Suggests MAGNET principles could be applied as a whole system approach to improve organisational cultures | Future research needs sound methodology, defined phenomena and underpinned by theory for organisational culture. Future research to use reliable tools, collaboration with experts in organisation change |
| Araujo et al., 2020 [12] | To systematically review and synthesize quantitative differences in health quality indicators before/ after accreditation or between accredited and non-accredited hospitals across seven healthcare quality dimensions | Large methodological differences between accreditation programmes. Research is challenging due to heterogenous settings. Unable to directly correlate accreditation as impacting outcomes, although effects appear mostly positive. Safety, efficiency and effectiveness are most commonly reported. Few studies report on patient-centredness, timeliness or access. No studies report on equity. | Can apply findings to assess relevance of accreditation processes | Utilisation of expert panels, future research into the impact of accreditation upon access, equity, timeliness, patient-centredness. Application of mixed methods advised. |
| Azagury et al., 2016 [24] | Systematic review of all available data about the link between bariatric surgery accreditation and surgical outcomes in the US. | Accredited / COE centres generally supported, but biases present | Nil | Nil |
| Avia & Hariati, 2019 [40] | To review and analyse the impact of hospital accreditation on the quality of care. | Accreditation has a positive impact on quality of care, with largest impact upon quality of management, then quality of results, then employee participation. | Nil | Research needs to examine the impact of hospital accreditation on the quality of nursing care. |
| Baidwan et al., 2020 [36] | A meta-analysis to assess the consistency of findings between COE accreditation for bariatric surgery and post-operative mortality and morbidity | Positive correlation found between accreditation and outcomes; however, this is not conclusive due to presence of heterogenicity and bias | Nil | Research needed on short- and long-term effects of accreditation on bariatric surgery outcomes. A large study conducting individual level analyses using multiple data sources that addresses some of the inconsistencies and weaknesses of the methods underlying the papers is required to generate conclusive evidence to make decisions regarding accreditation requirement |
| Brubakk et al., 2015 [25] | To systematically assess the effects of accreditation and/or certification of hospitals on both organizational processes and outcomes | Few studies specifically addressed the correlation between accreditation and certification of hospitals and patient outcomes, we could not reach firm conclusions regarding effective strategies in this area. The literature is dominated by descriptive studies attributing changes in the organization to the accreditation process | Nil | Research needed into clinical impact. Future investigations might control for case mix and time trends, employ suitable comparison groups, and consider other analytic approaches for analysing time series data such as interrupted time series data, or ARIMA methods. Interrupted time series analyses, Bayesian analysis and ARIMA may be suited for adjusting for clustering of effects within sites, while accounting for patient-level effects, and site-level structural measures. Studies addressing how and why the interventions might work, rather than just the effects of the intervention, might provide valuable information on complex interventions |
| Cerqueira, 2006 [19] | 1. Identify the benefits and challenges usually associated with accreditation  2. answer whether accreditation is an increasing practice among governments 3. summarise recommendations for improvement  4. provide recommendations for further research | Accreditation leads to improvement in quality of services | Best practice (accredited services) should be rewarded | Cost-benefit analysis, evaluation of accreditation |
| Danno et al., 2021 [35] | To analyse the knowledge produced regarding the practice environment in hospitals with quality improvement programs. | There is a paucity of high-quality controlled evaluations of the effectiveness and the cost-effectiveness of external inspection systems | Accreditation promotes a favourable nursing environment | Research needed within systems, comparing public, private and philanthropic institutions |
| Flodgren et al., 2011 [41] | To evaluate the effectiveness of external inspection of compliance with standards in improving healthcare organisation behaviour, healthcare professional behaviour and patient outcomes. | Insufficient primary research base to draw firm conclusions | Mandatory accreditation has benefits across a whole system vs voluntary | Further studies recommended, ideally Randomised Controlled Trials or Interrupted Time Series by design |
| Flodgren et al., 2016 [42] | To find out if external inspection of compliance with standards can improve improving healthcare organisation behaviour, healthcare professional behaviour and patient outcomes. | No change in number of eligible studies for inclusion, since 2011 | Accreditation is attractive to those running the health system, and so likely to continue | The review highlights the paucity of high‐quality controlled evaluations of the effectiveness and the cost‐effectiveness of external inspection systems. If policy makers wish to understand the effectiveness of this type of intervention better, there needs to be further studies across a range of settings and contexts and studies reporting outcomes important to patients. |
| Gamble et al., 2021 [31] | 1. What is being researched and written about the 'big picture' of hospital accreditation?  2. Currently, how do health accreditation processes in Australia demand, identify and measure evidenced, macro (policy) level quality and safety in maternity care? | No clear evidence for accreditation. Case for change is persuasive | Short- and medium-term structural changes to health accreditation systems are needed to prioritise, and mandate, continuity of midwifery care | nil specific, gaps identified |
| Greenfield & Braithwaite, 2008 [20] | To identify and analyse the research literature on accreditation. | Mixed views and inconsistent findings. Accreditation has been consistently found to promote change | Nil | Empirically grounded, comprehensive evidence is required |
| Hinchcliff et al., 2012 [43] | To examine accreditation's evidence-base and identify critical knowledge gaps regarding the development and evaluation of accreditation programmes | Findings generally present health service accreditation as a useful tool to stimulate quality improvement and processes. Quality of studies was moderate and modest in volume relative to global investment. Insufficiency of clinical outcomes measures to provide evidence of the impact of accreditation. | Nil | Quantitative studies may provide evidence of impact of processes, qualitative studies may contribute to theoretical developments as to how changes in quantitative indicators occur. Cost benefit analysis is needed for rational decision making in accreditation, policy development, as is the role of consumers. |
| Hovlid et al., 2020 [44] | To explore how external inspections can contribute to mediate change and improve the quality of care in healthcare organisations. | Most studies are descriptive and characterise the study population. No studies used and experimental design to test the mediators of change in organisations subject to external inspections. Inspections can affect different mediators of organisational change, and our findings can thereby enhance our understanding of why inspections seem to have varying effects. | Our findings can provide guidance for policy makers and inspectors on how future inspections should be designed and conducted to be more effective | Future studies should further explore relationships between how the inspections are carried out, their contextual setting and the way they can mediate change in care delivery in the inspected organisations. |
| Hussein et al., 2021 [26] | To identify and analyse the evidence on the impact of hospital accreditation. | Introducing hospital accreditation stimulates performance improvement and patient safety. | Efforts to incentivize and modernize accreditation are recommended to move towards institutionalization and sustaining the performance gains. | Utilizing longitudinal designs and controlling for exogenous confounders could help detect causal conclusions of accreditation effect |
| Johnston et al., 2020 [32] | What impact does Magnet designation have on emergency department nurses ‘outcomes? | Magnet accreditation suggested to influence collaboration among staff, broadly impactful at the organisational level, but the impact upon specialty areas is unclear | Magnet-like' Emergency Departments can enhance nursing staff satisfaction, reduce turn-over and enhance recruitment | More research needed into the impact of MAGNET accreditation on ED nursing, also upon patient care |
| Khan et al., 2021 [33] | What are the impacts of hospital accreditation in the three Middle East countries selected? (Iran, Jordan, Saudi Arabia) | Accreditation programs most likely promote improvements, processes, communication, and teamwork. Robust conclusions not possible, however, due to a lack of high-quality study designs | Organisational dynamics to be considered in the pursuit of hospital accreditation | Mixed-method or quasi-experimental studies, utilising objective and subjective data, may be the most suitable approach. Research gaps include impacts of hospital accreditation on patient safety indicators, patient satisfaction and experience, economic outcomes. |
| Kilsdonk et al., 2015 [45] | To assess the methodological characteristics of international studies on the impact of accreditation and external peer review on the quality of care in order to create a general research framework. | The evidence for the impact of accreditation and external peer review on the quality of care is still inconclusive | Accreditation programs focus on organisation and processes rather than outcomes, impact can be measured if programs have clear and measurable goals | Uniformity in research methods, use a simple framework, avoid cross sectional design |
| Lazzeri et al., 2019 [46] | 1. To explore what has been written on these topics in the Italian context  2. to analyse and discuss original experiences of accreditation and the results derived from them | Accreditation is a positive process to improve quality, however not all "quality" components are improved in the same way | Variability and the influence of external factors mean that quality standards should be periodically redefined | Studies should determine the minimum standards for health care quality and find new strategies that work with accreditation to ensure the highest level of quality |
| Mansour et al., 2020 [47] | To what extent are the structures and processes of hospital accreditation drawn from international models perceived as successful, and to what extent are they shaped by national policy contexts in low- and middle- income countries? | Accreditation is a tool for improvement, regulation, Universal Health Coverage, medical tourism. Accreditation has global and national influences, with donor agencies involved in low- and middle- income countries | Need to determine how to reduce administrative costs of accreditation | Future empirical research on accreditation underpinned by explicit theory, further research is also needed to study the background of some emerging policies, the role of international actors, and a cost-benefit analysis |
| Mumford et al., 2013 [21] | To determine whether economic techniques have been used to inform whether the benefits justify the costs | Few cost studies, but unable to find costs of remedial work post accreditation | A clearer definition of benefits is needed | A formal cost benefits analysis is required as a refence point against which accreditation reforms can be evaluated |
| Ng et al., 2013 [48] | To identify what factors could affect the successful implementation of an accreditation programme, and investigate the potential impact of the accreditation exercise on quality improvement in hospitals | Identified factors that may facilitate the successful implementation of accreditation - which align with those of change. Difficult to find evidence of accreditation effectiveness | Benefits of accreditation may include staff engagement, communication, MDT building, positive changes in organisational culture, and awareness of quality improvement. | Cost effectiveness evaluation, determine the correlation of accreditation results with indicators, |
| Petit dit Dariel & Regnaux, 2015 [27] | To identify the effect of a hospital's Magnet accreditation on nursing and patient outcomes | Mixed and inconsistent results surrounding the impact and effectiveness of Magnet accreditation on patient and nurse outcomes | Hospital and accreditation bodies should collaborate and call for more rigorous research designs | Longitudinal and quasi-experimental research recommended |
| Rodríguez-García et al., 2020 [49] | Compared with non-MAGNET hospitals, do MAGNET hospitals show different outcomes with regard to nurses, patients and health care organisations? | Perceptions of staff and patients is that MAGNET status improves outcomes. Indication that MAGNET status relates to better nursing, patient and organisational outcomes. | Nursing leaders are better informed as to whether to pursue MAGNET status or not | Longitudinal, mixed-methods approach suggested to determine if MAGNET related outcomes are sustained. Knowledge gap identified into the experience of nursing students. |
| Salmond et al., 2009 [28] | To examine the current evidence of the impact of Magnet designation on patient and nurse outcomes. Specifically, 1. What impact/ influence foes Magnet designation have on organisational outcomes including organisational climate or the professional work environment? 2. What impact/ influence does Magnet designation have on nursing outcomes including, but not limited to nursing satisfaction, recruitment and retention in acute care hospitals for the registered nurse? 3. What impact/ influence does Magnet designation have on patient outcomes including nurse-sensitive patient outcomes in acute care hospitals? 4. Does the economic investment for Magnet designation support the outcomes? | 1. Magnet hospitals have a significantly stronger professional nursing practice environment in comparison to non-Magnet, reputational Magnet and Magnet-Aspiring Hospitals. There is less burnout, more satisfaction 2. Magnet nurses perceive themselves to be more autonomous, and that Magnet hospitals are better resourced, with better management. There is no difference in nurse-physician relationships | 1. use evidence-based interventions.  2. measure nurse satisfaction and patient outcomes.  3. Evaluate financial implications of improved practicing environments and the potential return on investment. | Research needed to  1. Provide clarity on consistent metrics for patient outcomes that are influenced by nursing practice.  2. need to standardise measurements to facilitate meta-analysis.  3. evaluate process and outcomes at both the macro and microsystem levels.  4. research potential economic benefits |
| Swathi et al., 2020 [50] | To evaluate the effects of hospital accreditation. To compare the perception of different stakeholders towards the effects of hospital accreditation | Accreditation has a positive impact on health care organisation performance | Greater use of objective clinical outcomes and patient or consumer satisfaction measures would convince stakeholders in understanding the benefits of accreditation | Research should use mixed-methods and longitudinal design, possibly including quantitative outcome-based data, and relying upon qualitative data for aspects that cannot be quantitatively measured. Research into the disconnection between some health care professionals and perceptions of accreditation |
| Tabrizi et al., 2011 [29] | To identify the key attributes of major established health care accreditation systems and examine their relative advantages and disadvantages from the perspective of an emerging economy country. | Different accreditation programs have different aims, focus, design and maturity | Accreditation bodies should remain independent to governments and funders | Nil |
| Van Wilder et al., 2021 [22] | To assess the current evidence base on the impact of accreditation, public reporting and inspection on patient processes and outcomes | Most studies reported that accreditation had no observable impact on patient outcomes. | A Policy revision to determine the added value of the current system, aiming for a sustainable future system that benefits patients | Further research is required to study how inspections affect patient outcomes, healthcare professional perceptions of current policy, financial impacts of current policy. Implementation science needs to be considered to understand the contexts in which patient outcome improvements could be achieved. |
| Vist et al., 2009 [30] | To present all available documentation about the effect of certification and accreditation of hospitals | There is a lack of documentation to assess the effect of certification and accreditation of hospitals | Nil | There is a need for well-planned and controlled studies of the effect of certification and accreditation of hospitals |

Table 3

Table 4: Findings within the health care quality dimensions

| **Author and year** | **Findings within the health care quality dimensions** |
| --- | --- |
| Alkhenizan & Shaw, 2011 [37] | Effectiveness: suggestion that accreditation has positive effect on guidelines and processes.  Patient centredness: no effect.  Safety: accreditation associated with improved infection control, improved outcomes |
| Alkhenizan & Shaw, 2012 [38] | Efficiency: Concerns that accreditation is not efficient |
| Almasabi et al., 2014 [23] | Patient-centredness: no relationship found. |
| Almoajel, 2012 [39] | Effectiveness: use of clinical indicators encourages use of clinical guidelines |
| Anderson et al., 2018 [34] | Efficiency: staff retention  Patient-centredness: improves through improvements in nursing practice environment |
| Araujo et al., 2020 [12] | Effectiveness: mixed positive and null effect.  Efficiency: mostly positive findings.  Access: just one study found, null effect.  Patient-centredness: mixed null and positive.  Equity: nil findings.  Timeliness: limited to two studies/ null effect.  Safety: mostly positive findings |
| Azagury et al., 2016 [24] | Effectiveness: accreditation is associated with improved outcomes.  Efficiency: accreditation influences marshalling of resources.  Safety: accreditation is associated with post bariatric surgical complications |
| Avia & Hariati, 2019 [40] | Efficiency: accreditation benefits human resource allocation, teamwork, productivity.  Patient-centredness: accreditation may improve hospital infrastructure; cross department communication could improve patient experience.  Safety: accreditation improves written procedures, focus on falls prevention etc. |
| Baidwan et al., 2020 [36] | Effectiveness: lower mortality (interpret with caution)  Safety: accredited hospitals are associated with lower complication following bariatric surgery; however, this is to be interpreted with caution |
| Brubakk et al., 2015 [25] | Efficiency: concerns raised about financial and labour resources being diverted from clinical care, to achieve accreditation. |
| Cerqueira, 2006 [19] | Effectiveness: Accreditation is considered to promote best practice  Efficiency: accreditation influences the use of financial resources, however, also increases costs and workloads. Positive impact on staff turnover.  Patient centredness: accreditation has been found to have both no, and a positive effect on patient satisfaction indicators, and is considered to boost public confidence.  Safety: suggestion that accreditation reduces harm and infection, mixed results with respect to improved sanitation. Accreditation promotes patient safety processes, aimed at reducing risk |
| Danno et al., 2021 [35] | Efficiency: MAGNET accreditation attracts and retains nursing staff.  Patient centredness: MAGNET accreditation associated with higher levels of patient satisfaction |
| Flodgren et al., 2011 [41] | Nil |
| Flodgren et al., 2016 [42] | Nil |
| Gamble et al., 2021 [31] | Nil |
| Greenfield & Braithwaite, 2008 [20] | Effectiveness: inconclusive.  Patient-centredness: insufficient evidence, inconclusive.  Safety: inconclusive |
| Hinchcliff et al., 2012 [43] | Effectiveness: inconsistent results.  Efficiency: mixed suggestion that lower turnover in accredited organisations is related to satisfaction with processes resulting from accreditation, but accreditation is costly for human and financial resources.  Patient-centredness: insufficient evidence.  Safety: inconsistent results. |
| Hovlid et al., 2020 [44] | Efficiency: accreditation can be time consuming and redirect resources from clinical work.  Patient-centredness: inspection processes and recommendations should be more directed to patient care |
| Hussein et al., 2021 [26] | Effectiveness: positive effect on length of stay.  Efficiency: variable, found to increase productivity, reduce costs, but also place higher demands on staff and equipment expenditure. Other studies found no change in cost per case.  Patient-centredness: no evidence.  Safety: patient safety culture improved at the organisational level (i.e. incident reporting) |
| Johnston et al., 2020 [32] | Efficiency: MAGNET accreditation promotes lower staff turnover.  Timeliness: MAGNET accreditation may positively influence patient flow |
| Khan et al., 2021 [33] | Effectiveness: implied improvement in evidence-based care through improved data usage  Efficiency: implied reduced costs.  Patient-centredness: increased responsiveness to patient complaints  Safety: mixed results |
| Kilsdonk et al., 2015 [45] | Nil |
| Lazzeri et al., 2019 [46] | Patient centredness: mixed results |
| Mansour et al., 2020 [47] | Access: Accreditation has been used in low- and middle- income countries to achieve universal health care.  Equity: Used to implement universal health care in low- and middle- income countries |
| Mumford et al., 2013 [21] | Efficiency: inconclusive. |
| Ng et al., 2013 [48] | Patient centredness: driven by patient choice to attract patients |
| Petit dit Dariel & Regnaux, 2015 [27] | Effectiveness: mixed results, unproven  Efficiency: accreditation is time consuming and expensive  Safety: mixed results unproven |
| Rodríguez-García et al., 2020 [49] | Effectiveness: reduced length of stay, mortality  Efficiency: less staff turnover, burnout. Decreased costs of care.  Patient-centredness: MAGNET status found to improve patient satisfaction.  Safety: inconclusive as to whether MAGNET hospitals have fewer clinical incidents |
| Salmond et al., 2009 [28] | Effectiveness: unproven.  Efficiency: Magnet hospitals experience lower staff turnover.  Safety: unproven |
| Swathi et al., 2020 [50] | Effectiveness: positive effect i.e. reduced length of stay.  Efficiency: concerns that this is impaired by accreditation.  Patient- centredness: inconsistent findings.  Safety: positive effect. |
| Tabrizi et al., 2011 [29] | Effectiveness: Accreditation models are not strong on this aspect  Efficiency: Accreditation models are not strong on this aspect  Patient-centredness: Accreditation programs are not consistent in their emphasis on patient- centredness.  Safety: processes are geared towards ensuring safety. |
| Van Wilder et al., 2021 [22] | Effectiveness: not proven  Patient-centredness: not proven  Safety: not proven |
| Vist et al., 2009 [30] | Nil |

Table 4
