# Supplementary material for: Hospital accreditation: an umbrella review
Source: Int J Qual Health Care. 2023 Feb 4;35(1):mzad007. doi: 10.1093/intqhc/mzad007 (PMC9950788; doi:10.1093/intqhc/mzad007)
Supplement: mzad007_Supp [file mzad007_supp.zip › suppl_data/search_strategy.docx]

Search strategy

1. CINAHL (via EBSCOhost):

S1 hospital 575,932

S2 accreditation 18,344

S3 ‘literature review’ OR ‘systematic review’ OR ‘scoping review’ OR ‘narrative review’ OR ‘rapid review’ OR ‘meta-analysis’ 358,131

S4 S1 AND S2 AND S3 89

CINAHL (via EBSCOhost) results: **89**

1. Embase

#1 ‘hospital/exp’ OR hospital 9,897,805

#2 ‘accreditation’ 50,653

#3 ‘literature review’ OR ‘systematic review’ OR ‘scoping review’ OR ‘narrative review’ OR ‘rapid review’ OR ‘meta-analysis’ 793,700

#4 #1 AND #2 AND #3 386

Embase results: **386**

1. Medline (via EBSCOhost)

S1 hospital 6,358,548

S2 accreditation 32,448

S3 ‘literature review’ OR ‘systematic review’ OR ‘scoping review’ OR ‘narrative review’ OR ‘rapid review’ OR ‘meta-analysis’ 940,097

S4 S1 AND S2 AND S3 258

Medline (via EBSCOhost) results: **258**

1. PubMed

#1 Search: hospital 645,823

#2 Search: accreditation 4,038

#3 Search: ‘literature review’ OR ‘systematic review’ OR ‘scoping review’ OR ‘narrative review’ OR ‘rapid review’ OR ‘meta-analysis’ 337,801

#4 Search: ((hospital) AND (accreditation)) AND (‘literature review’ OR ‘systematic review’ OR ‘scoping review’ OR ‘narrative review’ OR ‘rapid review’ OR ‘meta-analysis’) 1,388

PubMed results: **1,388**

1. Scopus

1 TITLE-ABS-KEY (hospital) 2,377,040

2 TITLE-ABS-KEY (accreditation) 61,685

3 TITLE-ABS-KEY (‘literature AND review’ OR ‘systematic AND review’ OR ‘scoping AND review’ OR ‘narrative AND review’ OR ‘rapid AND review’ OR ‘meta-analysis’ 991,291

4 (TITLE-ABS-KEY (hospital)) AND (TITLE-ABS-KEY (accreditation)) AND (TITLE-ABS-KEY (‘literature AND review’ OR ‘systematic AND review’ OR ‘scoping AND review’ OR ‘narrative AND review’ OR ‘rapid AND review’ OR ‘meta-analysis’)) 279

Scopus results: **279**

1. Cochrane Database of Systematic Reviews

#1 ‘hospital’ 345,191

#2 ‘accreditation’ 501

#3 ‘literature review’ OR ‘systematic review’ OR ‘scoping review’ OR ‘narrative review’ OR ‘rapid review’ OR ‘meta-analysis’ 104,252

#4 #1 AND #2 AND #3 63

Cochrane Database of Systematic Reviews results: **63**

1. JBI EBP Database (via Ovid)

#1 hospital.mp. [mp=text, heading word, subject area node word, title] 2851

#2 accreditation.mp. [mp=text, heading word, subject area node word, title] 121

#3 (‘literature review’ or ‘systematic review’ or ‘scoping review’ or ‘narrative review’ or ‘rapid review’ or ‘meta-analysis’).mp. [mp=text, heading word, subject area node word, title] 276

#4 1 and 2 and 3 82

JBI EBP Database (via Ovid): **82**
