# Supplementary material for: Hospital accreditation: an umbrella review
Source: Int J Qual Health Care. 2023 Feb 4;35(1):mzad007. doi: 10.1093/intqhc/mzad007 (PMC9950788; doi:10.1093/intqhc/mzad007)
Supplement: mzad007_Supp [file mzad007_supp.zip › suppl_data/Supplementary_file_2.__Quality_Assessment.docx]

JBI Critical Appraisal Checklist for Systematic Reviews and Research Syntheses

| **Author** | **Title** | **Q1** | **Q2** | **Q3** | **Q4** | **Q5** | **Q6** | **Q7** | **Q8** | **Q9** | **Q10** | **Q11** | **Score** |
| --- | --- | --- | --- | --- | --- | --- | --- | --- | --- | --- | --- | --- | --- |
| Alkhenizan & Shaw, 2011 [37] | Impact of accreditation on the quality of healthcare services: a systematic review of the literature | N | U | U | Y | U | U | U | Y | N | Y | NA | 3Y 2N 5U 1NA |
| Alkhenizan & Shaw, 2012 [38] | The attitude of health care professionals towards accreditation: A systematic review of the literature | N | U | U | Y | NA | N | U | Y | N | Y | Y | 4Y 3N 3U 1NA |
| Almasabi et al., 2014 [23] | A systematic review of the association between healthcare accreditation and patient satisfaction | N | U | U | Y | N | NA | N | Y | N | Y | Y | 4Y 4N 2U 1NA |
| Almoajel, 2012 [39] | Relationship between accreditation and quality indicators in hospital care: A review of the literature | N | U | U | N | U | N | N | Y | N | NA | NA | 1Y 5N 3U 2NA |
| Anderson et al., 2018 [34] | Impact of MAGNET hospital designation on nursing culture: an integrative review | Y | Y | Y | Y | Y | Y | Y | Y | N | Y | Y | 10Y 1N |
| Araujo et al., 2020 [12] | Hospital accreditation impact on healthcare quality dimensions: a systematic review | N | U | U | Y | Y | U | Y | Y | Y | Y | Y | 7Y 1N 3U |
| Arzagury et al., 2016 [24] | Bariatric Surgery Outcomes in US Accredited vs Non-Accredited Centers: A Systematic Review | N | U | U | N | Y | Y | U | Y | N | Y | NA | 4Y 3N 3U 1NA |
| Avia & Hariati, 2019 [40] | Impact of hospital accreditation on quality of care: A literature review | N | U | U | N | NA | NA | N | Y | N | NA | NA | 1Y 4N 4NA |
| Baidwan et al., 2020 [36] | A meta-analysis of bariatric surgery-related outcomes in accredited versus unaccredited hospitals in the United States | N | U | U | N | Y | U | Y | Y | N | NA | Y | 4Y 3N 3U 1NA |
| Brubakk et al., 2015 [25] | A systematic review of hospital accreditation: the challenges of measuring complex intervention effects | Y | Y | Y | Y | Y | Y | Y | Y | Y | Y | Y | 11Y |
| Cerqueira, 2006 [19] | A literature review on the benefits, challenges and trends in accreditation as a quality assurance system | N | U | U | N | NA | N | N | Y | N | Y | Y | 3Y 5N 2U 1NA |
| Danno et al., 2021 [35] | Quality improvement programs and the professional nursing practice environment: an integrative review | Y | Y | Y | Y | Y | U | Y | Y | N | Y | N | 8Y 2N 1U |
| Flodgren et al., 2011 [41] | Effectiveness of external inspection of compliance with standards in improving healthcare organisation behaviour, healthcare professional behaviour or patient outcomes | Y | Y | Y | Y | Y | Y | Y | Y | Y | Y | Y | 11Y |
| Flodgren et al., 2016 [42] | External inspection of compliance with standards for improved healthcare outcomes | Y | Y | Y | Y | Y | Y | Y | Y | Y | Y | Y | 11Y |
| Gamble et al., 2021 [31] | Hospital accreditation: Driving best outcomes through continuity of midwifery care? A scoping review | Y | Y | Y | Y | NA | NA | NA | Y | NA | Y | Y | 7Y 4NA |
| Greenfield & Braithwaite, 2008 [20] | Health sector accreditation research: a systematic review. | NA | U | U | Y | NA | N | N | Y | N | NA | NA | 2Y 3N 2U 4NA |
| Hinchcliff et al., 2012 [43] | Narrative synthesis of health service accreditation literature | NA | U | U | Y | Y | Y | Y | Y | Y | Y | Y | 8Y 2U 1NA |
| Hovlid et al., 2020 [44] | Mediators of change in healthcare organisations subject to external assessment: a systematic review with narrative synthesis | N | U | U | Y | Y | Y | Y | Y | N | Y | Y | 7Y 2N 2U |
| Hussein et al, 2021 [26] | The impact of hospital accreditation on the quality of healthcare: a systematic literature review | N | U | U | Y | Y | Y | Y | Y | Y | Y | Y | 8Y 1N 2U |
| Johnston et al., 2020 [32] | What impact does Magnet designation have on emergency department nurses' outcomes? A scoping review | Y | Y | Y | Y | NA | NA | NA | Y | NA | Y | Y | 7Y 4NA |
| Khan et al., 2021 [33] | The impact of hospital accreditation in selected Middle East countries: a scoping review | Y | Y | Y | Y | NA | NA | Y | Y | NA | Y | Y | 8Y 3NA |
| Kilsdonk et al., 2015 [45] | Evaluating the impact of accreditation and external peer review | N | U | U | Y | N | N | U | Y | N | NA | Y | 3Y 4N 3U 1NA |
| Lazzeri et al., 2019 [46] | Accreditation and quality in the Italian national health care system: A 10 years’ long review | N | U | U | N | NA | N | N | Y | N | NA | N | 1Y 6N 2U 2NA |
| Mansour et al., 2020 [47] | The development of hospital accreditation in low- and middle-income countries: a literature review | N | U | U | Y | NA | N | U | Y | N | Y | Y | 4Y 3N 3U 1NA |
| Mumford et al., 2013 [21] | Health services accreditation: what is the evidence that the benefits justify the costs? | N | U | U | Y | Y | Y | Y | Y | Y | Y | Y | 8Y 1N 2U |
| Ng et al., 2013 [48] | Factors affecting the implementation of accreditation programmes and the impact of the accreditation process on quality improvement in hospitals: a SWOT analysis. | Y | Y | U | N | U | U | U | Y | N | Y | Y | 5Y 2N 4U |
| Petit dit Dariel & Regnaux, 2015 [27] | Do Magnet®-accredited hospitals show improvements in nurse and patient outcomes compared to non-Magnet hospitals: a systematic review | Y | Y | Y | Y | Y | Y | Y | Y | Y | Y | Y | 11Y |
| Rodríguez-García et al., 2020 [49] | How Magnet Hospital Status Affects Nurses, Patients, and Organizations: A Systematic Review: Findings support the pursuit of Magnet recognition | Y | Y | Y | Y | U | Y | Y | Y | U | Y | Y | 9Y 2U |
| Salmond et al., 2009 [28] | A comprehensive systematic review of evidence on determining the impact of Magnet designation on nursing and patient outcomes: is the investment worth it? | Y | Y | Y | Y | Y | Y | Y | Y | N | Y | Y | 10Y 1N |
| Swathi et al., 2020 [50] | Impact of accreditation on performance of healthcare organizations: A review of global studies | N | U | U | Y | NA | N | U | Y | N | Y | Y | 4Y 3N 3U 1NA |
| Tabrizi et al., 2011 [29] | Advantages and Disadvantages of Health Care Accreditation Models | N | U | U | Y | NA | N | N | Y | N | NA | NA | 2Y 4N 2U 3NA |
| van Wilder et al., 2021 [22] | Is a hospital quality policy based on a triad of accreditation, public reporting and inspection evidence-based? A narrative review | Y | Y | Y | Y | NA | N | Y | Y | N | Y | Y | 8Y 2N 1NA |
| Vist et al., 2009 [30] | Effect of Certification and Accreditation of Hospitals | N | U | U | Y | U | U | NA | NA | NA | NA | Y | 2Y 1N 4U 4NA |

^[[1]](#footnote-1)^

1. JBI Critical Appraisal Checklist for Systematic Reviews and Research Syntheses [16] questions

   ## **Is the review question clearly and explicitly stated?**

   ## **Were the inclusion criteria appropriate for the review question?**

   ## **Was the search strategy appropriate?**

   ## **Were the sources and resources used to search for studies adequate?**

   ## **Were the criteria for appraising studies appropriate?**

   ## **Was critical appraisal conducted by two or more reviewers independently?**

   ## **Were there methods to minimize errors in data extraction?**

   ## **Were the methods used to combine studies appropriate?**

   ## **Was the likelihood of publication bias assessed?**

   ## **Were recommendations for policy and/or practice supported by the reported data?**

   ## **Were the specific directives for new research appropriate?**

   [↑](#footnote-ref-1)
